# American Home Hospitals

**Published:** 1905-11-11

**Authors:** 


### HOSPITAL ADMINISTRATION.

### CONSTRUCTION AND ECONOMICS.

### AMERICAN HOME HOSPITALS.

#### THE COREY HILL HOSPITAL, BROOKLINE, BOSTON, MASS.

This institution contains thirty beds, and has been specially built and planned as a home hospital. It is beautifully situated on a hill overlooking Boston and Cambridge, Mass., and for hygienic reasons the site offers immense advantages. It has been organised by a number of physicians and surgeons, now numbering twenty-five, connected with Boston and the district, who have subscribed amongst them some $150,000—that is £30,000. They have placed the business control in the hands of two men of business, each being a partner in a large firm of solicitors. The plan of the institution is to give patients the opportunity of having one room or a suite of rooms with a bathroom to themselves, or the use of a bathroom in conjunction with one other patient. The rooms are charged at rates to suit all classes. Thus the charge for suites varies from $125 to $50 per week for two people. On the first floor the average rate for a single room is £8 8s.; on the second floor, £14; and on the third floor, £6 6s. per patient per week. On the latter floor there are two rooms at £4 and four at £5 per week per patient. These rates seem to us to be moderate, having regard to the excellence of the accommodation. Patients who wish it can take a suite of three rooms with a balcony, so that they can have a relative or friend, or a servant, with them during their residence in the home hospital.

The charges vary from $20 to $125 per week, in accordance with the accommodation selected, the average cost for rooms, nursing, food, etc., being about $50, or £10, a week per patient. If the case is a surgical one, a charge of $15 is made in addition for the use of the operation theatre, which includes the supply of all dressings, drugs, and other medical requisites. If a special nurse is required, the charge is $4 for each period of twelve hours, or $8 per diem when a special day and night nurse is employed.

When a patient pays $28 a week for special nursing, $21, or $3 per diem of this sum goes to the nurse, and $7 to the management. The ordinary remuneration of a nurse in a private family in the United States is $21, or four guineas per week.

#### Patients, Nurses and Accommodation.

During the first year, ended the 30th of June 1905, the Corey Hill Hospital admitted 360 patients, and 295 operations were performed.

The actual number of nurses on the staff is twenty-eight, for whom accommodation is provided in two houses rented for their use; but when the hospital is full forty nurses are sometimes employed. The management have made arrangements with five training-schools to supply a limited number of non-graduate probationers in their third year, who give their services to the institution and receive the advantage of instruction in the nursing of private cases. Of the twenty-eight nurses on the regular staff twelve are usually non-graduate nurses in their third year. The hospital contains thirteen beds on the ground floor, eight on the first, and nine on the second floor. The arrangements in all departments are most complete, and it is remarkable that a new building, probably the first of its kind in the world, should have been planned and opened where there are so few modifications which might be made with advantage. The whole of the floors with the exception of the operation theatre suite are made of polished wood. A year's experience once more proves that terrazzo pavement, which has been put down in each of the two operation theatres, and in the anæsthetising, sterilising, and recovery rooms attached, is not suitable for use in a modern hospital. Several cracks were apparent in this flooring, and having regard to the perfection aimed at by the management we have no doubt that they will replace it in a short time, for hygienic reasons, by teak floors of an improved pattern. The operation theatres are well planned, excellently lighted and arranged, and we were glad to find that they were relatively small in size, and that the management have had the wisdom to finish them with four coats of good oil paint; which, after all, is the best treatment, as experience proves, for every portion of a modern hospital operation theatre.

#### The Responsibility of Architects for Excessive Cost.

Seeing that the management have spared no expense, and that the cost per bed, including the site, of this hospital works out at something like $5,000 or £1,000; in view of the paper read by a leading architect at the recent hospital congress, advocating marble as the most suitable treatment for theatre walls, we have here definite proof that American surgeons of eminence favour the simplest and best treatment—*i.e.* four coats of paint for operation theatre walls. This is good evidence that the extravagance in the cost of operation theatres, which has grown to such proportions of late years as to be, in fact, a crying evil, must be charged to the account of the architects who are mainly responsible for the growing but needless and excessive cost of modern hospital construction.

Unless architects mend their ways it will be essential for the medical profession to take this matter up, and to insist that hospital managers shall be supported in resisting the architects, so as to prevent the cost of hospital construction from remaining at its present extravagant rate, which causes many generous and wealthy donors to refuse to give their money to hospitals for building purposes. We congratulate the eminent surgeons of Boston upon the object lesson they have given in the operation theatres at the Corey Hill Hospital, where money was practically no object, though provided by themselves; for it demonstrates in the most practical manner the necessity for curbing the extravagance of the modern architect. As we said on a recent occasion, any architect of repute who will give his mind and attention to the production of an efficient modern hospital building at a reasonable cost, will certainly make his fortune, and attain an additional reputation which should make him the envy of his professional brethren. Everywhere in New England the study of existing hospitals reveals the fact that the best results in regard to treatment are being attained in some of the cheapest buildings, often erected like the Moabit Hospital at Berlin for a temporary purpose, but which have been retained and used for decades of years because of their efficiency in practice. We are confident that the day will soon come, if it has not already arrived, when every hospital which is dependent upon public support for its maintenance must be so managed as to secure that its building expenditure shall in no instance exceed as a maximum £250, or $1,250 per bed.

### The Management Excellent.

The Corey Hill Home Hospital is under the management of Miss Stone, who acts as superintendent. Miss Stone was trained at the Pennsylvania Hospital, New York, has had a varied experience in hospital management in the United States, and is undoubtedly a most able, conscientious, and successful administrator. No one can visit this hospital, and carefully examine into every department as we had the privilege of doing on the day of our visit, without being struck with the efficiency which is everywhere apparent in every detail of each department. To mention one point out of many, the cold storage arrangements for the proper keeping of the food is so managed as to be practically perfect; especially when it is compared with similar departments in some larger hospitals in New England. Cold storage, unless it is supervised with considerable care and ability, makes the consumption of food often a task rather than a pleasure; and a visit to some cold storage meat stores must make the practised inspector of nuisances lose his appetite for several meals afterwards. Fish, we know, is sometimes a difficulty, but meat can be preserved for all the time it is right to retain it in a cold store, if adequate cleanliness and attention be given to the plant and store, so as to keep the atmosphere free from any taint of unpleasantness. This result is secured at the Corey Hill Hospital, to the great credit of Miss Stone and her assistants. We felt that it would be a pleasure to have any meal prepared for a patient in this hospital served to us, but this could not be said with truth of some of the larger hospitals which we have visited in the United States of America.

Miss Stone has evidently given a great deal of thought to many points in hospital management, and the condition of her linen store, and the arrangement of the lavatories—which, by the way, were too small for two nurses to work in at the same time—prove her knowledge of the subject and the excellence of her methods. We found in this hospital a stand for bed-pans, hot water bottles, and other similar articles, which might usefully find a place in every hospital lavatory. It is constructed of iron tubing, and is about five feet in height; it is roughly thirty inches long, eighteen inches wide, and has four shelves with an interval of twelve inches between each shelf. Each of these shelves consists of an iron grating, but it would undoubtedly be an improvement for at least half of the top shelf to be made solid.

### The Working and Resident Staff.

Miss Stone has the assistance of seven graduate nurses, and each floor is under the direct supervision of one such assistant. These assistants act for practical purposes as matrons in charge of their section of the hospital, and supervise the whole of the nursing arrangements and the actual work done by the nurses. There is a housekeeper who looks after the linen, the laundry and the servants. A dietitian who is a graduate of the Boston School of Cookery is responsible for the food and kitchen arrangements, and supplies the outlines of each day's menu which is settled by Miss Stone herself in conference with the dietitian. There are seventeen female and four male servants, and the hospital is in the medical charge of a resident physician. It will thus be seen that the staff is a large one, as it consists of some thirty people in addition to a nursing staff which varies from twenty-eight to forty, according to the number of patients in the hospital. Everything connected with the rooms is scrupulously clean and absolutely efficient. We do not remember ever inspecting a hospital which we have found in a more perfect condition than that of the Corey Hill Hospital at Boston. This fact is an honour to the medical profession and to everybody connected with it. Although the charges made may appear somewhat high, they can be regarded in no sense as excessive when it is remembered that everything in this institution is of the very best, that nothing is scamped or slurred over, and that everybody does, in fact, have the very best of everything always.

If a true judgment may be formed from the actual condition of matters as we found them to be on the 15th of October, 1905, this might appear to be excessive praise, did we not add that we spent upwards of two hours in the hospital, and examined everything, including the food, in detail, from top to bottom.

We regard Miss Stone as a fine specimen of the modern American nurse. She is a credit to her training school, and, we have no doubt, possesses deservedly the confidence of her employers, and the gratitude of every patient who may enter this hospital. Our visit gave us very real pleasure, because we found the Home Hospital in Boston, established as it is some twenty-nine years after the first institution of its kind, Fitzroy House, was started in London, working under identical regulations, and containing all the facilities which English experience proves to be desirable when the aim of the management is to afford a maximum of comfort and facility to private patients who are prepared to pay adequately for the best hospital accommodation when they have to undergo a serious operation.

